# The role of NEDD4 related HECT-type E3 ubiquitin ligases in defective autophagy in cancer cells: molecular mechanisms and therapeutic perspectives

**DOI:** 10.1186/s10020-023-00628-3

**Published:** 2023-03-14

**Authors:** Rui Zhang, Shaoqing Shi

**Affiliations:** 1 Department of Thoracic Surgery, The Seventh People’s Hospital of Chengdu, Chengdu, 610021 Sichuan People’s Republic of China; 2 Scientific Research Laboratory Center, First Affiliated Hospital of Kunming Medical University, Kunming, 650032 Yunnan People’s Republic of China

**Keywords:** Autophagy, Cancer, E3 ligase, NEDD4, Ubiquitin

## Abstract

The homologous to the E6-AP carboxyl terminus (HECT)-type E3 ubiquitin ligases are the selective executers in the protein ubiquitination, playing a vital role in modulation of the protein function and stability. Evidence shows the regulatory role of HECT-type E3 ligases in various steps of the autophagic process. Autophagy is an intracellular digestive and recycling process that controls the cellular hemostasis. Defective autophagy is involved in tumorigenesis and has been detected in various types of cancer cells. A growing body of findings indicates that HECT-type E3 ligases, in particular members of the neural precursor cell expressed developmentally downregulated protein 4 (NEDD4) including NEDD4-1, NEDD4-L, SMURFs, WWPs, and ITCH, play critical roles in dysregulation or dysfunction of autophagy in cancer cells. The present review focuses on NEDD4 E3 ligases involved in defective autophagy in cancer cells and discusses their autophagic function in different cancer cells as well as substrates and the signaling pathways in which they participate, conferring a basis for the cancer treatment through the modulating of these E3 ligases.

## Introduction

The E3 ubiquitin-protein ligases, the matchmakers in the ubiquitination cascade, are implicated in the regulation of various steps of the autophagic process, the major lysosome-dependent degradation pathway (Yin et al. 2020). Autophagy provides a homeostatic control mechanism and has been found to be defective in cancer (Russell and Guan 2022). Regarding the relevant clinical and therapeutic aspects of autophagy, there is emerging attentions in the exploring the responsible factors affecting the autophagy machinery in the diseases. By a comprehensive databases search, we found that, during recent years, there has been continuously growing evidence that shows a key role of HECT-type E3 ligases, particularly members of Neural precursor cell expressed developmentally downregulated protein 4 (NEDD4) family, in defective autophagy in cancer. Thus, the present review was conducted to address the following questions: (1) which members of the NEDD4 E3 ligase family are implicated in defective autophagy in cancer cells?, (2) which types of cancers are affected?, (3) what is their activity in autophagy in different cancer cells; autophagy inhibitor or autophagy inducer and tumor promoter or tumor suppressor ?, (4) what are their new substrates and molecular mechanisms underlying their effects?, (5) How can they be targeted to conquer different cancers?. To this end, the following sections were arranged. First, an overview of NEDD4 ubiquitin ligases as well as the autophagy process and its role in cancer cells were briefly presented in the following introduction subsections. Afterward, all published data regarding the role of NEDD4 ubiquitin E3 ligases in the autophagy process in cancer have been reviewed and discussed in detail.

### NEDD4 ubiquitin E3 ligases: a snapshot view of enzymatic activity and structure

E3 ubiquitin ligases are the selective executers in the protein ubiquitination and, thus, implicate in the two major protein degradation pathways, the ubiquitin–proteasome system (UPS) and autophagy (Yin et al. 2020). Ubiquitin is a highly conserved 76 amino acid globular protein. Ubiquitination is a reversible enzymatic conjugation event, which forms an isopeptide bond between the carboxyl group of C-terminal Glyc76 on ubiquitin and the ε-amino group of a Lysine residue on the substrate. Ubiquitin attachment onto target proteins includes a multistep reaction that needs the sequential and coordinated activity of a cascade of three enzymes: a ubiquitin-activating enzyme (E1), a ubiquitin-conjugating enzyme (E2), and a ubiquitin ligase (E3). The E3 ligases transfer activated ubiquitin to a Lysine residue on the target substrate through an interaction involving both the E2 conjugating enzyme and the substrate (Vere et al. 2020).

NEDD4 is a well-known family of the homologous to the E6AP carboxyl terminus (HECT)-type E3 ligases. NEDD4 family comprises nine E3 ligase members: NEDD4-1, NEDD4L, WW domain-containing E3 ubiquitin-protein ligase 1 (WWP1), WWP2, NEDL1 (HECW1), NEDL2 (HECW2), Smad ubiquitin regulatory factors (SMURF)1, SMURF2, and ITCH. NEDD4 members show a highly similar domain architecture consisting of an N-terminal protein kinase C-related membrane/lipid-binding C2 domain mediating attachment of NEDD4 E3 ligases to membrane compartments (Dunn et al. 2004; Plant et al. 2000; Angers et al. 2004; Kumar et al. 1997), two to four tryptophan-tryptophan (WW) domains located in the central part (N-terminus) for the substrate recognition and binding through the interaction with Proline-rich motifs (mainly PPxY) or phosphorylated Serine/Threonine-Proline regions on the target substrate (Kumar et al. 1997; Staub and Rotin 1996), as well as the catalytic HECT domain at the C-terminus (Kumar et al. 1997; Weber et al. 2019; Dodson et al. 2015). The HECT domain directly catalyzes the covalent bond between ubiquitin and target substrates through a two-step reaction: first, they capture the activated ubiquitin from E2 conjugating enzymes in a transthiolation reaction on their catalytic cysteine, and then, the ubiquitin moiety is transferred to a lysine on the substrate. The HECT domain is highly conserved and consists of N- and C-terminal lobes connected by a flexible linker chain. The N-lobe contains the E2-binding site (Fotia et al. 2006), while the C-lobe carries the active-site cysteine catalyzing the thioester bond with the ubiquitin moiety (Verdecia et al. 2003; Huang et al. 1999). The flexible linker permits the C-lobe to move around and assist the ubiquitin transfer from the E2 to the E3 (Weber et al. 2019). In basal status, the NEDD4 E3 ligases can be kept in a catalytically inactive state through an autoinhibitory conformation in which the N-terminal domains (either the C2 or WW domains) interact with the C-terminal HECT domain to luck the HECT activity and prevent substrate or E2 access (Wan et al. 2011; Wiesner et al. 2007; Wang et al. 2019; Zhou et al. 2014).

Proteins can be modified by mono-ubiquitination, as a result of the attachment of a single ubiquitin, or by polyubiquitination through the sequential attachment of ubiquitin moieties on lysine residues. Ubiquitin contains seven internal lysine residues (K6, K11, K27, K29, K33, K48, and K63) that can accept another ubiquitin molecule in subsequent rounds of ubiquitination, finally generating multiple types of polyubiquitin chains (Vere et al. 2020; Xu et al. 2009). Mono- or polyubiquitination and the exact composition of linkage chain determines the distinct fate of the substrates. For example, K63-linked poly-ubiquitylated and mono-ubiquitylated substrates are preferentially degraded by the autophagy/lysosome system, whereas K48-linked ubiquitination is mainly believed to target substrates for proteasome degradation (Kwon and Ciechanover 2017). Of note, K63-linked and K48-linked polyubiquitination compete with each other to activate autophagic proteins in response to stress conditions or to degrade them when the stress situation is resolved, respectively. In particular, K48-polyubiquitination-mediated degradation of autophagy proteins is necessary to terminating the autophagy response (Yin et al. 2020).

### An overview of autophagy

The intracellular protein homeostasis is majorly controlled by using two pathways of protein degradation, the UPS and autophagy. Whereas the UPS is the main cellular pathway to degrade short-lived proteins, autophagy is the fundamental catabolic mechanism for degrading and recycling damaged organelles as well as long-lived proteins, protein aggregates, and protein complexes. Autophagy is a conserved self-digestion process, through which cytosolic constituents are sequestered by lipid bilayer vesicles and subsequently transferred to lysosomes for degradation (Cao et al. 2021).

Autophagy exists in a basal (constitutive) as well as stimulated state. Basal autophagy occurs in most cells and tissues under normal physiological conditions to maintain cellular homeostasis. Basal autophagy is also responsible for cellular architectural alterations that happen during development and differentiation (Adelipour et al. 2022; Hu et al. 2019). However, stimulated autophagy can occur in response to cellular stresses such as nutrient or growth factor starvation, high temperature, overcrowding, hypoxia, endoplasmic reticulum (ER) stress, and microbial infection (Cao et al. 2021). In response to such stresses, autophagic degradation is activated to provide biosynthetic demands, reprogram cellular metabolism, and permit cell viability. Notably, starvation induces nonselective autophagy that engulfs any cytosolic constituents. Starvation-induced autophagy permits the cell to recycle nutrients from digested organelles and proteins, thereby maintaining the cellular biosynthetic capacity via providing amino acids for de-novo protein synthesis, and preserving the cellular energy source (ATP) by supplying free fatty acids and amino acids for the Krebs cycle. However, selective autophagic degradation acts by recognition and targeting specific cellular material, such as protein aggregates (aggrephagy), injured organelles (mitophagy for the mitochondria disposal, ERphagy for the ER disposal, and pexophagy for the perioxisomes disposal), as well as intracellular pathogens (xenophagy) (Lamark and Johansen 2021; Janssen et al. 2021).

The autophagy process consists of an orderly set of events: the initiation, phagophore nucleation, sequestration and autophagosome formation, the fusion of the autophagosome with the lysosome, and cargo digestion and recycling. The promotion of autophagy is started with the nucleation of a sequestering membrane, creating a cup-shaped phagophore, which stems from lipid bilayers provided majorly by the ER, but also by endosomes and the Golgi apparatus. A part of cytosol, including organelles, is subsequently engulfed by the elongating phagophore to create a double-membrane vesicle called the autophagosome. Eventually, the outer membrane of the autophagosome merges with the lysosomal membrane to create an autolysosome compartment, where engulfed cytosolic components are degraded by the acidic lysosomal hydrolases (Cao et al. 2021; Sidibe et al. 2022).

The core autophagic machinery relies on autophagy-related (ATG) proteins, which assemble into functional complexes that are recruited to autophagy membrane compartments and work in sequential order to deliver the cytosolic cargo to the lysosomes (Zhou et al. 2022). The master regulator of autophagy is the mammalian target of rapamycin complex 1 (mTORC1), which inhibits autophagy by suppressing the activity of Unc-51-like kinase 1 (ULK1) (Dossou and Basu 2019; Ganley et al. 2009; Hosokawa et al. 2009; Jung et al. 2009; Noda and Fujioka 2015). ULK1 is a serine/threonine kinase and one of the most upstream ATG proteins required for the initiation steps of autophagy in mammalian cells. Under stressful conditions, AMP-activated protein kinase (AMPK) suppresses mTORC1 and activates ULK1 that forms a stable protein kinase complex with autophagic proteins ATG13, ATG101, and FIP20 to initiate autophagy (Dossou and Basu 2019).

The activated ULK complex localizes to discrete sites on the ER and induces the phagophore nucleation via phosphorylating components of the class-III phosphatidylinositol 3-kinase (PI3KC3) complex comprising Beclin-1, Vps34/PI3K, Vps15, ATG14L, UV resistance-associated gene (UVRAG), and Rubicon. Upon phosphorylation, the PI3KC3 complex induces local production of phosphatidylinositol-3-phosphate (PI3P) at ER structures termed omegasomes, where the effector proteins are recruited to initiate the phagophore nucleation (Karanasios et al. 2016).

**Fig. 1:**
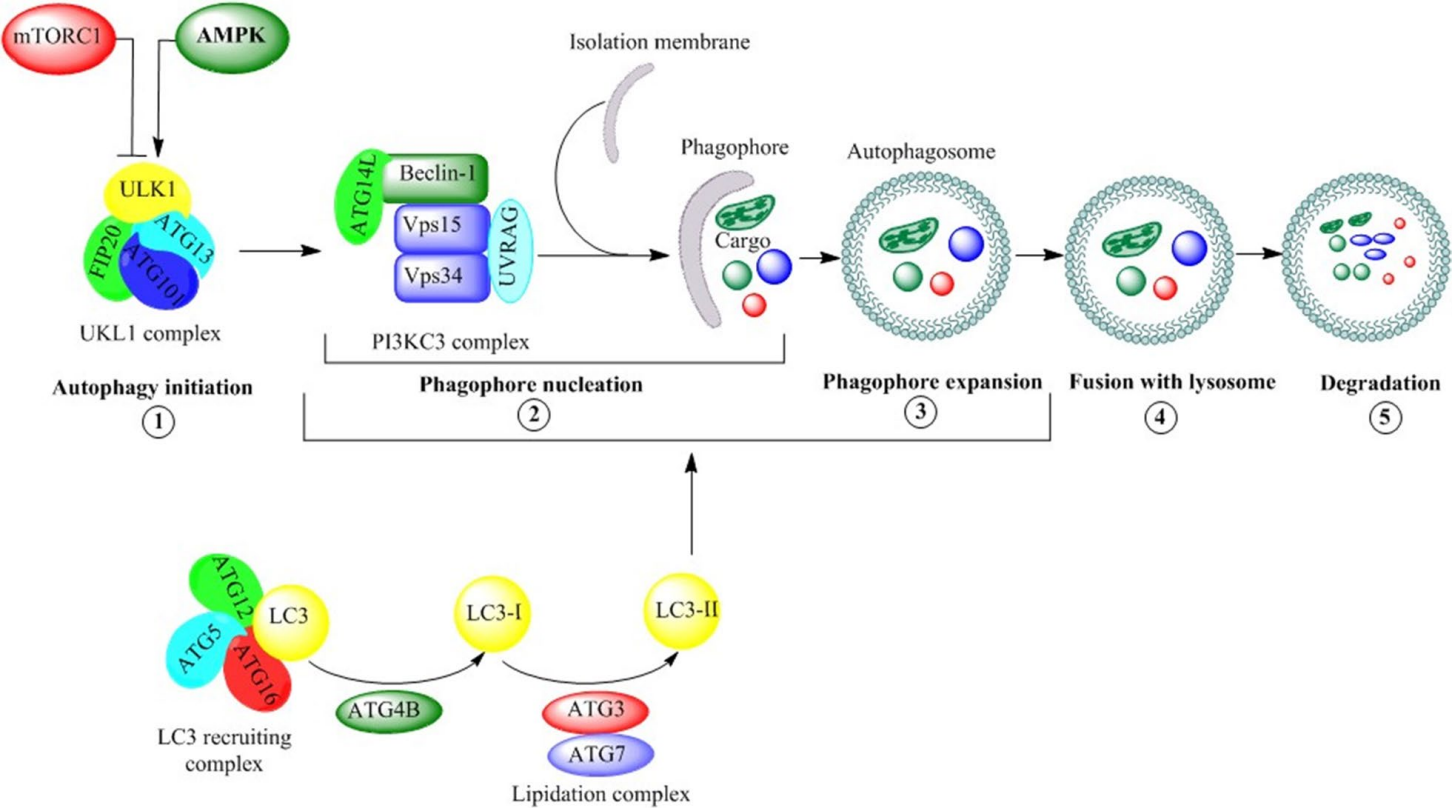
The schematic view of the role of NEDD4 E3 ubiquitin ligases in the autophagic process in cancer cells

ATG proteins orchestrate the elongation and expansion of the phagophore membrane to the autophagosome. The ATG5 ~ ATG12-ATG16L complex recruits ATG8 [microtubule-associated protein 1 light chain 3 (LC3)] protein that is subsequently lipidated by the sequential activity of ATG4B as well as ATG7 and ATG3 (Carlsson and Simonsen 2015). The lipid conjugation process is initiated via converting LC3 to the active cytosolic isoform LC3-I by the protease activity of ATG4B, in which ATG4B cleaves LC3 to expose the C-terminal Glycine for the subsequent lipidation reaction. ATG3/ATG7 mediate conjugation of membrane-associated phosphatidylethanolamine (PE) and convert LC3-I to lipidated LC3-II (Fig. 1). Indeed, LC3-II is a membrane-anchored form of LC3 that is essential for phagophore elongation and for facilitating the specific recruitment of cargos in selective autophagy (Kabeya et al. 2000; Walker and Ktistakis 2020).

For the selective autophagic degradation, specific autophagy adaptors (receptors), such as p62 [also called sequestosome 1 (SQSTM 1)] (Pankiv et al. 2007; Zheng et al. 2009; Wurzer et al. 2015), autophagy and Beclin 1 Regulator 1 (AMBRA1) (Rita, et al. 2018), Optineurin (OPTN), nuclear dot protein 52 kDa (NDP52) (Heo et al. 2015; Mostowy et al. 2011; Thurston et al. 2009; Muhlinen et al. 2012), and neighbor of BRCA1 gene (NBR1) (Walinda et al. 2014; Riley et al. 2010; Kirkin et al. 2009), attach a ubiquitin-tagged cargo to a nascent autophagosome by concurrently binding the cargo and LC3-II on the sequestering membrane. These adaptors possess a ubiquitin-associated domain (UBA) and an LC3-interacting region (LIR) allowing their binding to ubiquitinated cargoes and LC3-II, respectively (Wurzer et al. 2015; Kirkin et al. 2009; Long et al. 2010; Isogai et al. 2011). Indeed, autophagy adaptors act not only as a bridge between ubiquitinated cargoes and LC3-II by the UBA domain but also as a transporter for cargo delivery to autophagosomes by the LIR domain.

### The search strategy

A systematic literature search was performed in electronic databases, including Web of Science, PubMed, Scopus, and Google Scholar, without any language restrictions, to find all published articles dealing with the aims of the present study. The search was performed using the terms [(autophagy) AND (E3 ubiquitin ligase) AND (“HECT” OR “homologous to the E6AP carboxyl terminus” OR “NEDD4” OR “Neural precursor cell expressed developmentally downregulated protein 4”) AND (“cancer” OR “tumor”)] in titles and abstracts. In addition, the references of enrolled studies were also manually checked to find other related publications that were potentially missed from database searching.

### The role of NEDD4 E3 ubiquitin ligases in the autophagy process in cancer

Although autophagy can maintain the normal physiological function of cells, excessive autophagy can lead to diseases. The dysregulation of autophagy has been found to exert a role in various human diseases, such as neurodegenerative disorders (Fleming et al. 2022), rheumatic diseases (Celia et al. 2022), muscular diseases, cardiovascular diseases (Gatica et al. 2022), and cancer (Gundamaraju et al. 2022; Ariosa et al. 2021). Autophagy has been known to be a double-edged sword in cancer biology, acting both as a protector of cancer cell survival and a tumor suppressor depending on the cancer type and stage of cancer development. On the one hand, autophagy suppresses tumor growth in the early stages by inhibiting the proliferation of pre-cancerous cells, scavenging toxic molecules correlated with tumorigenesis, and removing damaged organelles. On the other hand, autophagy is able to induce tumor growth and survival in later stages. Tumors are under highly stressful conditions such as hypoxia and nutrient deprivation, and autophagy can increase stress tolerance and provide nutrients to meet the metabolic demands of cancer cells, thereby enhancing cancer-cell survival (Gundamaraju et al. 2022; Ariosa et al. 2021).

In addition, a fine-tuning of autophagic activity is important for the appropriate cellular hemostasis and growth, and defective autophagy with either excessive or low activity can cause cancer cell formation. Accumulating findings indicate that the dysregulation of NEDD4 family E3 ligases can be one of the molecular mechanisms attributed to the dual role of autophagy in cancer cells. A systematic search in different electronic databases indicated that, among HECT-type E3 ligases, members of the NEDD4 family including NEDD4-1, NEDD4L, SMURF-1, SMURF-2, WWP1, WWP2, and ITCH have been investigated in many various cancers with defective autophagy, as reviewed in next sections.

### Autophagic-mediated roles of NEDD4-1 in cancer cells

**Fig. 2:**
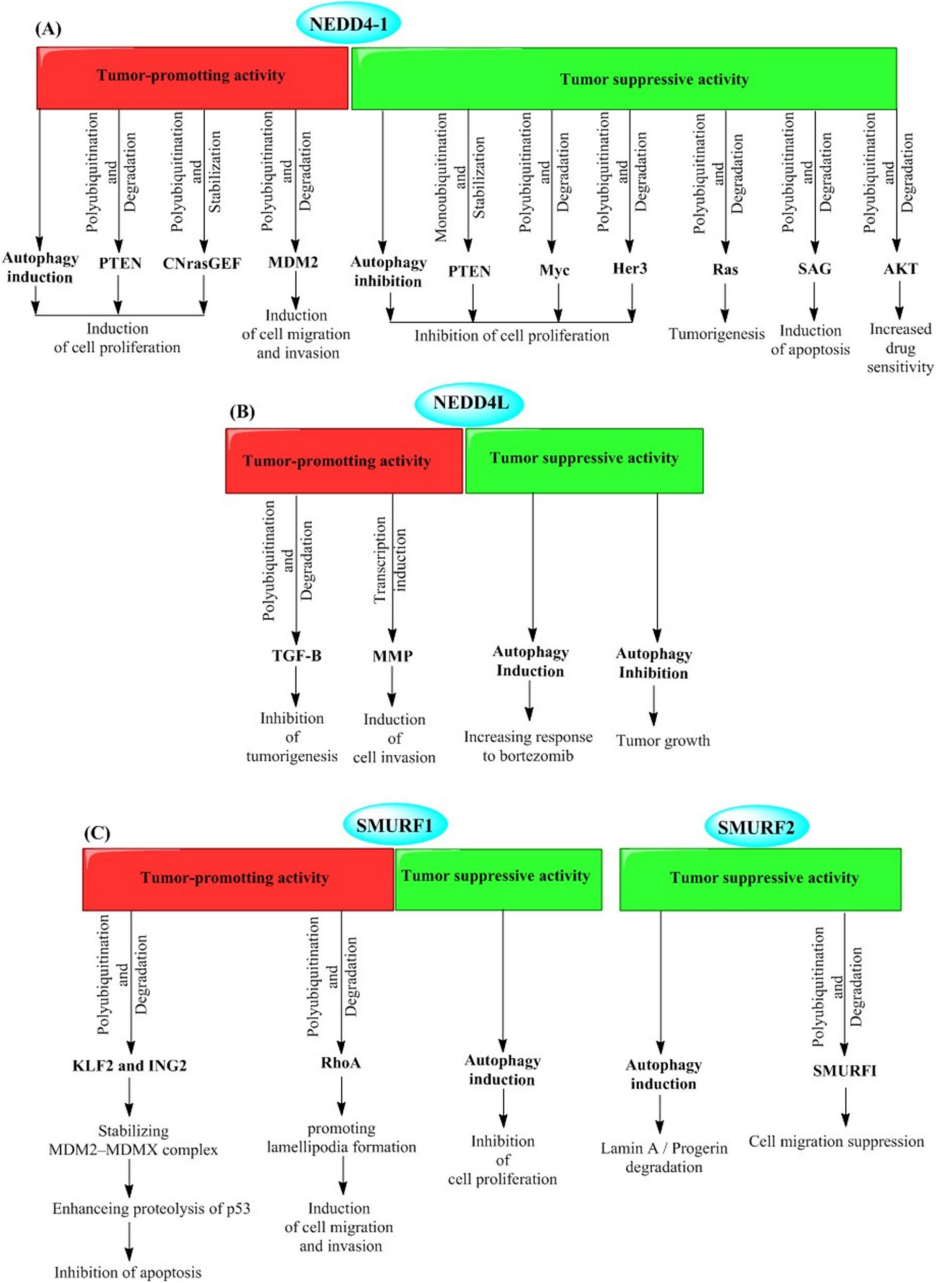

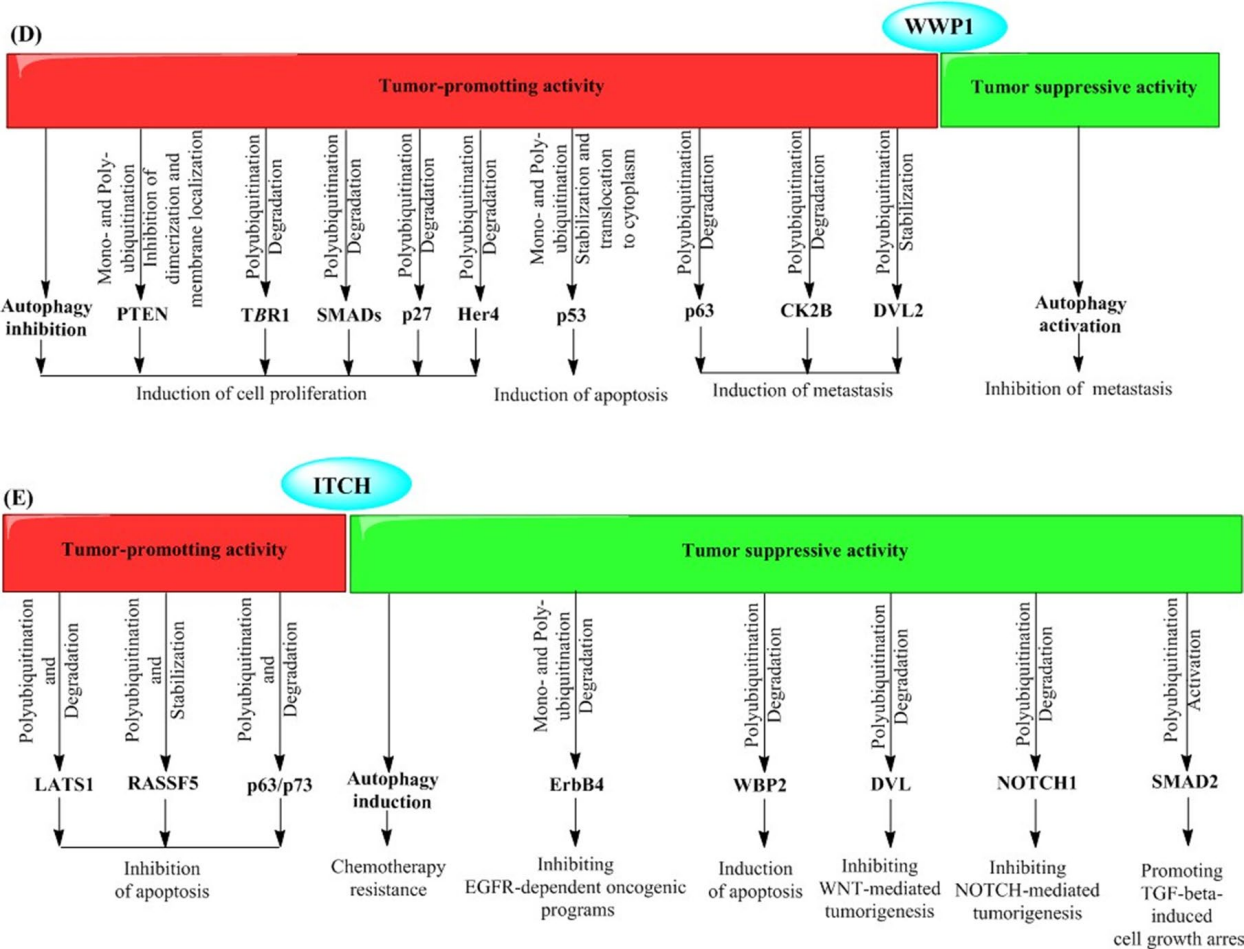
Molecular targets and pathways involving tumor promoter and tumor suppressive activities of NEDD4 E3 ubiquitin ligases, including NEDD4-1 (**A**), NEDD4-L (**B**), SMURFs (**C**), WWP1 (**D**), and ITCH (**E**)

The ubiquitin E3 ligase NEDD4-1 has been found to involve in the proliferation, migration, invasion, and drug sensitivity of cancer cells. NEDD4-1 exerts the dichotomous roles as an oncoprotein (Eide et al. 2013; Amodio et al. 2010; Wang et al. 2007; Xu et al. 2015; Huang et al. 2017; Li et al. 2015; Sun et al. 2017; Kim et al. 2008a; Jung et al. 2013; Singh et al. 2011; Verma et al. 2017; Yim et al. 2009) and a tumor suppressor (Zhou et al. 2014; Trotman et al. 2007; Liu et al. 2013; Huang et al. 2015; Huang et al. 2020a; Zeng et al. 2014; Platta et al. 2012) in cancer cells (Fig. 2A). The expression of NEDD4-1 has been reported to be elevated in several types of cancers including colorectal (Eide et al. 2013; Kim et al. 2008a), gastric (Kim et al. 2008a), breast (Jung et al. 2013; Singh et al. 2011; Verma et al. 2017; Yim et al. 2009), non-small-cell lung carcinoma (Amodio et al. 2010), bladder, prostate, cervical (Wang et al. 2007; Li et al. 2015), hepatocellular carcinoma (HCC) (Huang et al. 2017), and glioma (Zhang et al. 2013). Its oncogenic or tumor suppressor activities are mainly mediated by ubiquitination of proteins with oncogenic or tumor suppressor functions such as PTEN (Amodio et al. 2010; Wang et al. 2007; Kim et al. 2008a; Jung et al. 2013; Singh et al. 2011; Yim et al. 2009), MDM2 (Xu et al. 2015), CNrasGEF (Zhang et al. 2013; Pham and Rotin 2001), N-Myc and C-Myc (Liu et al. 2013), Her3 (Verma et al. 2017; Huang et al. 2015), SAG (Zhou et al. 2014), AKT (Huang et al. 2020a; Fan et al. 2013), and Ras (Zeng et al. 2014). In mechanism, the dual role of NEDD4-1 in cancer cells is ascribed to its non-selective ability to interact with the Proline-rich motifs that are universal regions in many proteins with different activities (Huang et al. 2019).

Growing evidence has shown that NEDD4-1 can also play the role in the autophagy process in cancer cells, thereby affecting tumor growth. Although some conflicting results have been published on the role of NEDD4-1 in the regulation of autophagy, the most of available investigational evidence appears to imply the pro-autophagic activity of NEDD4-1 in basal and starvation-promoted autophagy (Li et al. 2015; Sun et al. 2017; Pei et al. 2017; Xie et al. 2020) as well as selective autophagy such as mitophagy (Sun et al. 2017), infection-promoted autophagy (Pei et al. 2017), and aggrephagy (Lin et al. 2017; Xie et al. 2022).

Li et al. found that the overexpression of NEDD4-1 in cancer cells, such as the lung and prostate, can promote autophagy initiation by inhibiting mTORC1 to protect cancer cell survival. Notably, the knockdown of NEDD4-1 strongly elevated the active levels of mTOR protein, and as a result, inhibited autophagy activation and proliferation in lung and prostate cancer cells, supporting the association of elevated levels of NEDD4-1 with the induction of the protective autophagy in cancer cells (Li et al. 2015).

NEDD4-1 has been also found to regulate other steps of the autophagy process, including the phagophore nucleation and elongation as well as the substrate selection during mitophagy. NEDD4-1 can positively regulate the phagophore nucleation via targeting Beclin-1 in cancer cells (Sun et al. 2017; Pei et al. 2017). NEDD4-1 was detected to promote K6- and K27-conjugate polyubiquitination of Beclin-1, resulting in increased stability of Beclin-1 and elevated autophagy (Pei et al. 2017), which is consistent with the finding that showed the knockdown of NEDD4-1 caused a significant reduction of Beclin-1 protein and the inhibition of protective autophagy in cancer cells (Sun et al. 2017). Further investigations revealed that vacuolar protein sorting 34 (VPS34) is the other target of NEDD4-1 in the autophagy machinery and plays an important role in the NEDD4-1-mediated ubiquitination of Beclin-1 in cancer cells (Xie et al. 2020). VPS34 is the catalytic subunit of the PI3KC3 complex, which interacts with autophagic proteins such as Beclin-1 at the phagophore assembly sites to form functional complexes activating the phagophore formation (Mizushima 2018). Of note, the existence of VPS34 appears to be indispensable for the Beclin-1 stabilization. Interestingly, NEDD4-1 undergoes auto-ubiquitination that serves it as a scaffold for engaging the ubiquitin-specific protease 13 (USP13) to form a NEDD4-1/USP13 deubiquitination complex, which subsequently deubiquitinates and stabilizes VPS34 to induce phagophore nucleation in cancer cells. On the other hand, VPS34 depletion in lung cancer cells was found to not only impair the activity of NEDD4-1 to stabilize Beclin-1 (Xie et al. 2020), but also cause NEDD4-1-mediated proteasomal degradation of Beclin-1 via K11-linked ubiquitination (Platta et al. 2012). This suggests that the VPS34/Beclin-1 complex formation is essential for the NEDD4-1-mediated K6- and K27-linked ubiquitination and stabilization of Beclin-1. Thus, it can be proposed that VPS34, after activation by NEDD4-1, forms a complex with Beclin-1 and subsequently presents it to NEDD4-1 for K6- and K27-linked ubiquitination.

The NEDD4-1 can also involve in the phagophore elongation and substrate selection in cancer cells through interaction with LC3 and the autophagy receptor SQSTM1. Notably, the contribution of NEDD4-1 to autophagy was first explored by Behrends et al. through the network organization of the human autophagy system which revealed an interaction between NEDD4-1 and LC3 (Behrends et al. 2010). The subsequent study by Li et al. showed that downregulation of NEDD4-1 in prostate and lung cancer cells could reduce cancer cell proliferation and inhibit autophagy, which was accompanied by a reduction in the formation of LC3-positive phagophores and a decreased conversion of LC3-I to LC3-II (Li et al. 2015). Supportingly, further investigations by Sun et al. indicated that the knockdown of NEDD4-1 in HCC, neuroblastoma, and lung cancer cells caused the aberrant aggregation of the LC3 puncta and the presence of deformed mitochondria as well as reduced autophagosome biogenesis, which appeared as a severe defect in the activation of starvation-induced autophagy or mitophagy. Indeed, NEDD4-1 can directly interact with LC3 through a conserved LC3-binding motif in a region located between the WW and the C2 domains, thereby forming a functional complex and positively regulating autophagy at the phagophore elongation step (Sun et al. 2017). It is noteworthy that the interaction of NEDD4-1 with LC3 was found to be not only for association with autophagosomes (Sun et al. 2017) but also for activation of this E3 ligase to interact with and polyubiquitinate SQSTM1 (Sun et al. 2017; Lin et al. 2017). The NEDD4-1 dependent-polyubiquitination of SQSTM1 was detected to be mainly through K63 linkage (Lin et al. 2017), which is necessary for the SQSTM1-mediated selective autophagy (such as mitophagy), but not for its proteasomal degradation (Lin et al. 2017; Kim et al. 2008b; Johansen and Lamark 2011; Rogov et al. 2014; Stolz et al. 2014).

Thus, it can be concluded that, in cancer cells highly expressing NEDD4-1, such as prostate, HCC, neuroblastoma, and lung, NEDD4-1 can mediate starvation-induced autophagy and mitophagy, by inducing autophagosome biogenesis and stabilizing autophagy receptor SQSTM1, to protect cancer cell survival and growth.

On the other hand, a low expression of NEDD4-1 in cancer cells, like melanoma, was found to be associated with the activation of the immunoglobulin-containing and Proline-rich receptor-1 (IGPR-1)–mediated autophagy (Sun et al. 2021). IGPR-1 is a cell adhesion molecule that is overexpressed in some cancer cells and induces autophagy with a remarkable implication for tumor growth and angiogenesis (Sun et al. 2021; Rahimi et al. 2012; Woolf et al. 2017; Amraei et al. 2020). Activation of IGPR-1 by AKT/protein kinase B (PKB) and inhibitor of nuclear factor kappa-B kinase subunitβ (IKKβ) can induce autophagy at the initiation step through a mechanism involving activation of AMPK (Amraei et al. 2020; Ho et al. 2019). Sun et al. showed that increased expression of NEDD4-1 in melanoma cells can suppress IGPR-1–induced autophagy and tumor growth, by promoting K63- and K48-linked polyubiquitination and lysosomal degradation of IGPR-1 (Sun et al. 2021).

Therefore, a well-regulated NEDD4-1 can induce or halt autophagy in favor of cellular hemostasis. When its expression or activity is dysregulated, NEDD4-1 can cause protective autophagy in favor of the cancer cell requirement (Table 1).

**Table 1:** The target substrates of NEDD4 E3 ubiquitin ligases in the autophagic process in cancer cells

E3 ligase	Expression level	Cancer types	Molecular target	Autophagy Step	Effect on cancer cells
NEDD4-1	High	Prostate, HCC, Lung, Neuroblastoma	mTORC1 (−)	Initiation (+)	Protective
			VPS34 (+) Beclin-1 (+)	Phagophore nucleation (+)	Protective
			LC3-I (+) SQSTM1 (+)	Phagophore elongation (+) Substrate selection (+)	Protective
	Low	Melanoma	IGPR-1 (−)	Initiation (−)	Inhibitive
NEDD4L	Low	Multiple myeloma	mTORC1 (−) LC3-I (−)	Activating autophagy (autophagy step was not defined)	- Inhibitive - Involving in drug response
	Low	Pancreas	ULK1 (−) ASCT2 (−)	Phagophore nucleation (−)	Inhibitive
SMURF1	Not defined	HCC	UVRAG	Phagophore nucleation ( +)	Inhibitive
WWP1	High	AML	Not defined	Autophagosome formation (−)	Protective
	Low	Melanoma	KLF5 (−) BAP1 (−)	Initiation (+)	Inhibitive
WWP2	Not defined	Not defined	NDP52 (+) OPTN (+) SQSTM1 (+)	Selection (+)	Not defined

### Autophagic-mediated roles of NEDD4L in cancer cells

Accumulating investigations have reported different roles of the ubiquitin E3 ligase NEDD4L in cancer biology in various types of malignancies (Fig. 2B). NEDD4L exerts a tumor suppressive role and is correlated with poor prognoses in cancer cells where its expression is low, such as multiple myeloma (MM) (Huang et al. 2022), non-small cell lung carcinoma (Sakashita et al. 2013), malignant gliomas (He et al. 2012), and gastric cancer (Jiang et al. 2019; Gao et al. 2012). On the other hand, NEDDL also shows an oncogenic activity with a highly elevated expression in cutaneous T-cell lymphoma (Booken et al. 2008), gallbladder cancer (by modulating the transcription of matrix metalloproteinase genes *MMP-1* and *MMP-13*) (Takeuchi et al. 2011), prostate carcinoma (by ubiquitination and degradation of TGF-β) (Hellwinkel et al. 2011), and melanoma (Kito et al. 2014) [by ubiquitination and degradation of melanosomal transmembrane protein Melan-A/MART-1 (Lévy et al. 2005)].

Novel evidence recently reported by Huang et al. shows that NEDD4L favors the tumor suppressive function and involves in the drug sensitivity in the MM cancer cells, by acting as an autophagy activator (Huang et al. 2022). It was indicated that MM cells express low levels of NEDD4L and that the low NEDD4L expression by malignant plasma cells is a risk factor in MM patients (Huang et al. 2022). Notably, the low expression of NEDD4L was found to intensify bortezomib resistance in vitro and in vivo mainly due to autophagy impairment (Huang et al. 2022). Clinical and molecular assessments revealed that increased expression of NEDD4L coincided with autophagy activation, which was correlated with a significantly elevated probability of responding to bortezomib, a prolonged response duration, and improved overall prognosis in MM patients (Huang et al. 2022). Further investigations showed that NEDD4L induces autophagosome biogenesis by reducing the activated levels of mTOR and increasing the LC3-II/-I ratios as well as inhibiting proteasome-dependent protein degradation via binding the 19S proteasome and limiting its proteolytic function (Huang et al. 2022). Of note, the 19S proteasome is the regulatory subunit of the 26S proteasome that degrades ubiquitinated proteins and is a responsible factor for bortezomib resistance (Barrio et al. 2019; Oerlemans et al. 2008). To sum up, these findings, in addition to representing NEDD4L as a newly recognized autophagy activator, suggest both the application of NEDD4L as a new biomarker for predicting MM patients’ response to bortezomib as well as the usefulness of NEDD4L activators in combination with bortezomib as a novel therapeutic approach for the MM treatment. The autophagic activity of NEDD4L was further supported by another study that indicated NEDD4L mediates ER stress-induced autophagy as well as autophagy induced by both basal and nutrient starvation conditions in cultured cervical cancer cells and in the mouse (Wang et al. 2016). Especially, it was shown that, upon ER stress, NEDD4L is upregulated by the spliced form of X-box binding protein 1 (sXBP1), a transcription factor that regulates the expression of a variety of genes essential for recovering ER activity after ER stress (Wang et al. 2016).

On the other hand, findings reported by Lee et al. indicated that pancreatic cancer cells with low levels of NEDD4L predominantly relied on autophagy activation, which was associated with tumor growth and survival in both in vitro and in vivo studies (Lee et al. 2020a). Overexpressed NEDD4L was found to repress autophagy in pancreatic cancer cells by decreasing cellular levels of the autophagy protein ULK1 (Lee et al. 2020a; Nazio et al. 2016) and the glutamine transporter ASCT2 (Lee et al. 2020a). The interesting finding was that ASCT2, stabilized by the loss of NEDD4L, exerted an essential role in activating autophagy in NEDD4L-downregulated pancreatic cancer cells, in addition to its direct activity in delivering key substrates for mitochondrial metabolism (Lee et al. 2020a). Of note, it was shown that NEDD4L, through inhibiting autophagy, impaired mitochondrial metabolism by disrupting the cellular oxygen consumption rate (OCR), the mitochondrial membrane potential (MMP), and mitochondrial morphology to ultimately suppress pancreatic tumor growth. Notably, the low expression of NEDD4L in pancreatic cancer cells caused ULK1- and ASCT2-mediated autophagy as well as ASCT2-mediated glutamine uptake to provide adequate fuel to activate mitochondrial metabolism and enhance mitochondrial functional integrity, thereby preventing cell death and further facilitating the tumor growth (Lee et al. 2020a). The suppressive role of NEDD4L in autophagy is further supported by the Nazio et al. study that showed NEDD4L mediates K27- and K29-linked polyubiquitination and subsequent proteasome degradation of ULK1, thereby inhibiting phagophore formation and autophagy response (Nazio et al. 2016) (Table 1).

### Autophagic-mediated roles of SMURFs in cancer cells

SMURF1 and SMURF2, a SMURF1-related protein, are members of the NEDD4 family, which regulate several biological networks such as “transforming growth factor-β” and “bone morphogenetic protein” signaling pathways (Zhu et al. 1999; Lin et al. 2000; Kavsak et al. 2000; Ebisawa et al. 2001; Tajima et al. 2003; Murakami et al. 2003). These E3 ligases act in multiple biological processes including cell growth and differentiation, cell adhesion and migration, cell polarity, as well as autophagy, whereby participate in various physiological functions including bone formation, embryogenesis, and cancer development (Fig. 2C) (Shimazu et al. 2016; Sahai et al. 2007; Li et al. 2016; Fukuchi et al. 2002; Sato et al. 2022; Yu et al. 2020).

SMURF1 and SMURF2 have been found to be critical determinants of basal autophagy (Feng et al. 2019) and selective autophagy including mitophagy and xenophagy (Orvedahl et al. 2011; Borroni et al. 2018a; Franco et al. 2017); however, underlying molecular mechanisms are not clearly known. Growing evidence indicates that the autophagic activity of SMURFs plays the important role in cancer biology.

Recently, Feng et al. reported that ubiquitination of UVRAG, an important regulator of mammalian cell autophagy, by SMURF1 induces phagophore nucleation and suppresses the HCC growth (Feng et al. 2019). SMURF1 was found to recruit and directly interact with UVRAG through the PPxY motif, and subsequently catalyze the K29- and K33-linked polyubiquitination of UVRAG at K517 and K559 residues (Feng et al. 2019). Interestingly, it was revealed that SMURF1-mediated ubiquitination does not target UVRAG for proteasomal or lysosomal degradation, but positively regulates the autophagic activity of UVRAG (Feng et al. 2019). Indeed, ubiquitination of UVRAG at K517 and K559 by SMURF1 impedes its interaction with Rubicon (Feng et al. 2019) that negatively regulates the maturation of autophagosomes through binding to the UVRAG-PI3KC3 complex (Nakajima et al. 2017; Wang et al. 2015). Rubicon interacts with the catalytic subunit of PI3KC3, thus suppressing its lipid kinase activity. In sum, UVRAG ubiquitination inhibits Rubicon-PI3KC3 interaction, boosts the PI3KC3 activity, and, ultimately, induces phagophore nucleation (Feng et al. 2019). The ubiquitination of UVARG by SMURF1 was detected to be significantly elevated in HCC cells cultured in a glucose-depleted medium, showing that SMURF1 can mediate starvation-induced autophagy in HCC cells (Feng et al. 2019). Subsequent in vitro and in vivo studies indicated that UVRAG ubiquitination by SMURF1 could significantly facilitate the phagophore formation and lysosomal degradation of the epidermal growth factor receptor (EGFR), decrease the EGFR signaling, and suppresses proliferation of HCC cells and tumor growth in mice (Feng et al. 2019). The inhibitory effect of SMURF1 on HCC initiation and progression can be supported by another study that showed the negative impact of SMURF1 on the role of “melanoma cell adhesion molecule” in maintaining the transformative phenotype of HCC cells (Tang et al. 2015). Besides, SMURF1 has been also found to act as an autophagy receptor whereby interacts with hepatic lipid droplets and ER via its C2 membrane-binding domain and targets them for degradation by the autophagy pathway (Orvedahl et al. 2011) (Table 1).

The E3 ubiquitin ligase SMURF2 is another NEDD4 family member that plays a controversial role in cancer biology; some studies have indicated a tumor suppressive role of SMURF2 (Sato et al. 2022; Yu et al. 2020; Fukunaga et al. 2008; Zhang et al. 2015a; Chandhoke et al. 2016), whereas others reported its tumor-promoting role (Fukuchi et al. 2002; David et al. 2014). The autophagy-mediated tumor suppressor role of SMURF2 is supported by findings that show SMURF2 can bind and ubiquitylate Lamin A and its disease-correlated mutant variant Progerin (Borroni et al. 2018b). Lamin A is a major structural component of the nuclear lamina, and mutations in its encoding gene and/or changes in its expression levels lead to cancer and a variety of distinct degenerative diseases (Borroni et al. 2018b). Of note, histopathological evaluation of microarray data from several human cancer tissues, including breast cancer, breast invasive ductal carcinoma, and prostate cancer, indicated a reciprocal association between elevated levels of SMURF2 and reduced levels of Lamin A, and vice versa (Borroni et al. 2018b). Notably, subsequent investigations revealed that SMURF2-mediated ubiquitination of Lamin A and Progerin induces their disposal through the autophagic degradation pathway, in a tissue-specific manner (Borroni et al. 2018b). Accordingly, the abovementioned findings suggest that the tissue-specific autophagic activity of SMURF2 may be responsible for its controversial role in cancer development.

### Autophagic-mediated roles of WWPs in cancer cells

The ubiquitin E3 ligase WWP1 is another member of the NEDD4 family showing an important role in the maintenance and development of cancer, acting as either oncoprotein or tumor suppressive (Fig. 2D) (Kuang et al. 2021; Huang et al. 2020b). WWP1 is commonly overexpressed and genetically amplified in multiple types of human cancers, such as breast cancer (Kuang et al. 2021; Chen et al. 2007a; Nguyen Huu et al. 2008), prostate adenocarcinoma (Kuang et al. 2021; Chen et al. 2007b; Lee et al. 2019), gastrointestinal cancers (Kuang et al. 2021; Zhang et al. 2015b; Zhang et al. 2015; Cheng et al. 2014; Lin et al. 2013; Chen and Zhang 2018), as well as acute myeloid leukemia (AML) (Kuang et al. 2021; Sanarico et al. 2018) where acts as an oncoprotein E3 ligase. WWP1 has been reported as a component of the UPS system ubiquitinates several numbers of proteins and regulates various cellular processes such as protein degradation and trafficking as well as cell signal transductions such as the EGFR, TGF-β, PI3K-AKT, and WNT (Kuang et al. 2021; Huang et al. 2020b). This E3 ligase induces tumorigenesis by modulating post-translational stability or functions of various substrates with the tumor suppressive activity, such as PTEN (Lee et al. 2019; Lee et al. 2020b), TGF-β type 1 receptor (Komuro et al. 2004), SMADs (Morén et al. 2005; Seo et al. 2004), p63 (Li et al. 2008; Chen et al. 2018), p53 (Laine and Ze 2007), p27^kip^ (Sanarico et al. 2018; Cao et al. 2011), and HER4/ErbB4 (Feng et al. 2009; Li et al. 2009) as well as those with the tumor-promoting activity such as CK2β (Kim et al. 2018) and DVL2 (Nielsen et al. 2019; Zhao et al. 2021).

Of note, WWP1 was recently found to inhibit the autophagy signaling in AML cells where it is highly expressed (Sanarico et al. 2018). Sanarico et al. (2018) showed that WWP1 knockdown in AML cells induced autophagy by promoting autophagosome formation. Notably, autophagy activation upon WWP1 depletion inhibited the growth and proliferation of AML blasts and delayed leukemia progression in mice bearing AML cancer, while the ectopic expression of WWP1 accelerated the growth of AML cells. Shortly after WWP1 inactivation, the autophagosome formation in leukemic cells was detectable by the elevated conversion of LC3-I into the lipid-bound LC3-II as well as by accumulation of the autophagy-associated protein ATG7 and the reduced levels of the autophagy receptor SQSTM1, indicating an elevated autophagic turnover (Sanarico et al. 2018). Another piece of evidence showing localization of this E3 ligase in the main nucleation sites of the autophagosome maturation such as the endosomes, plasma membrane, and Golgi apparatus (Chen et al. 2008) further supports such a negative impact of WWP1 on the early steps of autophagy (Sanarico et al. 2018). Thus, WWP1 can prevent autophagosome building in AML by interfering with the degradation and/or function of proteins contributed to the phagophore nucleation and elongation steps. Mechanistically, however, it remains unknown how WWP1 modulates autophagy and whether ATG7 and LC3 may be WWP1 targets during autophagy activation. Nevertheless, although further research is warranted to elucidate the related substrates of WWP1 in the regulation of the autophagy process, the current findings suggest this E3 ligase is an important negative regulator of autophagy in AML.

On the other hand, there is evidence showing that WWP1 can act as the autophagy activator and tumor suppressor E3 ligase in skin cells (Jia et al. 2021), by ubiquitination and inducing proteasomal degradation of oncoprotein KLF5 (Human Kruppel-like factor 5) transcription factor (Jia et al. 2021; Chen et al. 2005). Notably, KLF5 is de-ubiquitylated by BRCA1 associated protein-1 (BAP1) (Jia et al. 2021; Qin et al. 2015) and suppresses autophagy by activating the PI3K-AKT-mTOR signaling (Jia et al. 2021) that can inhibit the autophagy initiation and the autophagosome formation via modulating the activity of AMBRA1 and ULK1 complex (Xu et al. 2020; Nazio et al. 2013). Recently, Jia et al. (2021) showed that the WWP1- BAP1-KLF5 axis is dysregulated in melanoma cancer; WWP1 was lowly expressed in melanoma cells and tissues whereas KLF5 and BAP1 were highly expressed and closely related to tumor metastasis. In melanoma patients, the expression level of WWP1 was positively associated with good prognosis whereas the expression levels of KLF5 and BAP1 were found to be positively associated with poor prognosis (Jia et al. 2021). Subsequent investigations showed that WWP1 overexpression in melanoma cells could mediate K48-linked ubiquitination of KLF5 and induce its proteasomal degradation, whereas BAP1 overexpression reverted this modification and increased KLF5 protein expression (Jia et al. 2021). Notably, overexpressed KLF5 could suppress melanoma cell autophagy by activating the PI3K-AKT-mTOR pathway, thereby inducing melanoma cell malignant phenotypes in vitro as well as progression and metastasis of melanoma in vivo (Jia et al. 2021) (Table 1). The contrary autophagic roles of WWP1 in AML and melanoma cancers can be due to different cellular contexts with different signaling networks regulating WWP1 or affected by WWP1.

WWP2 is another member of the NEDD4 family, most closely related to WWP1 and ITCH, and to a more limited extent to NEDD4-1 and NEDD4L (Weber et al. 2019; Lee et al. 2019; Chen and Matesic 2007). Several ubiquitination substrates have been reported for WWP2, including the tumor suppressor PTEN (Chen et al. 2016; Maddika et al. 2011), the RNA-editing enzyme ADAR2 (Marcucci et al. 2011), the catalytic subunit of RNA polymerase II (Caron et al. 2019; Li et al. 2007), the signaling protein I-SMAD7 (Soond and Chantry 2011), as well as the EGR2 (Chen et al. 2009) and OCT4 transcription factors (Xu et al. 2004). It was also recently reported that WWP2 can ubiquitinate autophagy receptors NDP52, OPTN, and SQSTM1, by which induces autophagy, particularly mitophagy (Jiang 2022). These autophagy receptors can selectively deliver cargo to the autophagosome through their binding to the LC3 protein that presents as a phospholipid conjugate with the autophagosome membrane (Farré and Subramani 2016; Kirkin and Rogov 2019; Heo et al. 2019) (Table 1). To the best of our knowledge, the effect of the autophagic role of WWP2 in cancer cells has not been investigated and are needed to be evaluated in future research.

### Autophagic-mediated roles of ITCH in cancer cells

The ubiquitin E3 ligase ITCH also called atrophin-1 interacting protein 4 (AIP4), has been found to involve in regulating immunological responses (Aki et al. 2015) and cancer progression (Yin et al. 2020b). A mounting body of reports exhibits the multifaceted oncogenic and tumor suppressor functions of ITCH in different human malignancies (Fig. 2E), due to its dynamic and context-dependent role in tumor cells (Yin et al. 2020b). This arises from the versatility of ITCH in mediating both proteolytic (K48) and non-proteolytic (K63, K27, and K33) ubiquitination of a growing list of tumor-related substrates (Yin et al. 2020b), such as the p53 family members p63 and p73 (Rossi et al. 2005; Rossi et al. 2006; Melino et al. 2006; Bellomaria et al. 2010; Browne et al. 2011; Bernardini et al. 2008), the tumor suppressor RASSF5/NORE1 (Suryaraja et al. 2013), the large tumor suppressor 1 (LATS1) (Ho et al. 2011; Salah et al. 2011), the lysosomal-associated protein multispanning transmembrane 5 (LAPTM5) (Ishihara et al. 2011), the epithelial kinase receptor ErbB4 (Sundvall et al. 2008), NOTCH1 (Qiu et al. 2000), SMAD2 (Bai et al. 2004), and the Wnt/β-catenin signaling pathway (Goto et al. 2020).

Interestingly, there are also independent reports (Chastagner et al. 2006; Marchese et al. 2003; Chhangani et al. 2014; Rossi et al. 2014) that, when taken together, confer evidence for an autophagic-mediated oncogenic role of ICTH in cancer cells. The first evidence of the ITCH autophagic activity can be concluded from the reports that showed the involvement of ITCH in lysosomal degradation of target substrates including Deltex and the chemokine receptor CXCR4, through their polyubiquitination (Chastagner et al. 2006; Marchese et al. 2003). Another finding that can reinforce an implication of ITCH in the autophagy process is a finding that revealed ITCH recruits denatured cytosolic proteins as well as components of the autophagy machinery such as LC3 and SQSTM1 (Chhangani et al. 2014). Further supporting is the results of high throughput screening of ITCH inhibitors that indicated clomipramine, an antidepressant drug earlier found to impede autophagy by blocking autophagolysosomal fluxes (Rossi et al. 2009), could specifically interact with the HECT domain of ITCH and irreversibly block its ubiquitination activity, coinciding with inducing autophagosome accumulation and autophagy inhibition (Rossi et al. 2014). In addition, ITCH has been detected to be over-expressed in various human cancers such as ovarian, breast, sarcomas (Salah et al. 2011), and anaplastic thyroid carcinoma (Ishihara et al. 2008), and ITCH depletion could potentiate the effect of chemotherapeutic drugs (Rossi et al. 2014). Consistently, the results of treating a panel of breast, prostate, and bladder cancer cell lines showed that clomipramine, concomitant with ITCH suppressive activity, could also synergize with chemotherapeutics in killing tumor cells, supporting the role of ITCH-dependent autophagy in cancer progression (Rossi et al. 2014).

Altogether aforementioned findings manifest a pro-autophagic function of ITCH in cancer cells, where its expression is high and can promote cell growth. However, further proof-of-concept studies are needed to clarify the exact molecular mechanisms underlying the autophagic activity of ITCH in cancer cells expressing high levels of ITCH. Nevertheless, available data suggest the ITCH-dependent autophagy as a novel mechanism in tumorigenesis.

### Therapeutic perspectives of NEDD4 targeting in cancer

Targeting ubiquitin enzymes has been found to be an attractive therapeutic strategy for cancer treatment (Beurden-Tan et al. 2017). Since E3 ubiquitin ligases determine substrate specificity, their targeting seems to be a more specific approach compared to E1/E2 enzymes. There have been findings that show HECT domains of E3 ligases provide druggable targets for cancer treatment (Mund et al. 2014; Quirit et al. 2017). For instance, experimental studies indicated that pan-HECT inhibitors, heclin and indole-3-carbinol derivatives, could suppress the HECT E3 ligase activity of the NEDD4 family enzymes and, thereby, decrease cancer cell proliferation and growth (Lee et al. 2019; Mund et al. 2014; Quirit et al. 2017). However, as explained in the last sections, each member of the NEDD4 E3 ligase family indicates multi-faced roles, pro- or anti-autophagic and tumor-suppressor or –promotor, in various types of cancer cells. Thus, strategies for specific modulation, induction or inhibition, of each NEDD4 E3 ligase may be more suitable for cancer treatment than strategies using pan-HECT inhibitors.

NEDD4-1 has been found to induce autophagy and proliferation in the prostate, HCC, neuroblastoma, and lung cancer cells (Li et al. 2015; Sun et al. 2017; Pei et al. 2017), suggesting that NEDD4-1 inhibition is a suitable therapeutic strategy for treating these cancer cells. However, NEDD4-1 is also relevant for ubiquitination and lysosomal degradation of pro-autophagic oncoprotein IGPR-1 and, as a result, for inhibiting the autophagy process and proliferation in melanoma cancer cells (Sun et al. 2021). Thus, NEDD4-1 inducers can be useful treatments for IGPR-activated cancer cells such as melanoma.

Further, NEDD4L was found to inhibit ULK1- and ASCT2-mediated mitophagy whereby suppressing adequate fuel supplementation via mitochondrial metabolism, leading to inducing cell death and inhibiting tumor growth in pancreatic cancer cells (Lee et al. 2020a). NEDD4L can also exert pro-autophagic and tumor suppressor activities in multiple myeloma and cervical cancer cells and increases the drug sensitivity in multiple myeloma (Huang et al. 2022). These findings show the usefulness of NEDD4L inducers as a promising therapeutic approach for the treatment of pancreatic, multiple myeloma, and cervical cancers.

Autophagy induction via SMURF1-mediatd activation of UVRAG was shown to functionally result in autophagic degradation of oncoprotein EGFR and, consequently, suppress HCC cell proliferation and tumor growth (Feng et al. 2019). SMURF1 also shows tumor-promotor activity in breast cancer cells where the ubiquitination function of SMURF2 can promote SMURF1 degradation and, consequently, may rescue important tumor suppressive substrates of SMURF1 to prevent malignant migration of tumor cells (Fukunaga et al. 2008; Borroni et al. 2018b). Thus, SMURF1 and SMURF2 inducers can be considered as effective treatments for HCC and breast cancer, respectively.

The WWP1 downregulation was found to induce autophagy activation which, in turn, suppresses the growth and proliferation of AML blasts and delays leukemia progression in mice bearing AML cancer (Sanarico et al. 2018), showing that WWP1 inhibition can be a valuable therapeutic approach for AML cancer. Ubiquitin-mediated degradation of oncoprotein KLF5 by WWP1 could induce autophagy signaling, thereby inhibiting melanoma cell malignant phenotypes in vitro as well as progression and metastasis of melanoma in vivo (Jia et al. 2021). Therefore, WWP1 inducers can be accounted for the therapeutic treatment of melanoma cancer. In the case of ITCH, the pro-autophagic and oncogenic roles in breast, prostate, and bladder cancer cells have been found and, thus, suggest ITCH inhibitors as the potential therapeutic tool in these cancer (Rossi et al. 2014).

To sum up, therapeutic inhibition or induction of each NEDD4 E3 ligase should be decided based on the type of cancer. Besides, a combination treatment by NEDD4-1 and WWP1 inducers for melanoma cancer, NEDD4-1 and ITCH inhibitors for prostate cancer, NEDD4-1 and SMURF1 for the HCC, as well as SMURF2 inducers and ITCH inhibitors for the breast cancer can be considered as the effective therapeutic approaches.

## Concluding remarks

- NEDD4-1 can govern both stabilization and degradation of target substrates via catalyzing different K-linkage types of ubiquitin chains, including K6 and K27 (for stabilizing Beclin-1), K11 (for proteasomal degradation of Beclin-1), K63 and K48 (for lysosomal degradation of IGPR-1), and K63 (for stabilizing SQSTM1).
- In prostate, HCC, neuroblastoma, and lung cancer cells, NEDD4-1 is highly expressed and favors tumorigenesis and positively regulates autophagy initiation via the inhibition of mTORC1, the phagophore nucleation via stabilization of VPS34-Beclin-1 complex, the phagophore elongation via interaction with LC3-I, as well as cargo selection via stabilization of the autophagy receptor SQSTM1. However, NEDD4-1 depicts the tumor suppressive and anti-autophagic roles in melanoma cancer cells where it negatively regulates autophagy via destabilizing IGPR-1.
- NEDD4L catalyzes K27- and K29-linked ubiquitination of ULK1 for proteasomal degradation.
- In MM cancer cells, NEDD4L favors a tumor suppressive role and positively regulates autophagy initiation via the inhibition of mTORC1 as well as the phagophore elongation via increasing lipidation of LC3-I to LC3-II. NEDD4L also acts as a tumor suppressor and negatively regulates autophagy initiation via destabilizing ULK1 and ASCT2.
- SMURF1 stabilizes UVRAG by catalyzing the K29- and K33-linked ubiquitination at K517 and K559 residues.
- In HCC cells, SMURF1 shows a tumor suppressive role and positively regulates the phagophore nucleation by enhancing the PI3KC3 activity via stabilization of UVRAG. SMURF1 also acts as an autophagy receptor whereby targets hepatic lipid droplets and ER through a mechanism involving its C2 membrane-binding domain.
- WWP1 exerts the autophagy inhibitor and the tumor promotor roles in AML cancer cells where it negatively regulates the phagophore nucleation and elongation likely via targeting ATG7 and LC3. WWP1 also shows the pro-autophagic and tumor suppressive activities in melanoma cells through mechanisms remaining unknown yet.

## Conclusion

Regarding the versatility of NEDD4 E3 ubiquitin ligases in catalyzing both proteolytic and non-proteolytic ubiquitination of a wide range of target substrates, their role in defective autophagy in cancer cells can be concluded to be context-dependent.

## Data Availability

The authors declare that there are no conflicts of interest and financial support for the present review article.
